# SAF-SD: Self-Distillation Object Segmentation Method Based on Sequential Three-Way Mask and Attention Fusion

**DOI:** 10.3390/s26072170

**Published:** 2026-03-31

**Authors:** Biao Wang, Jun Su, Volodymyr Kochan, Lingyu Yan

**Affiliations:** 1 School of Information Engineering, Wuhan College, Wuhan 430212, China; 2 Key Laboratory of Green Intelligent Computing Network in Hubei Province, Hubei University of Technology, Wuhan 430068, China; 3 Department of Information and Computing Systems and Control, West Ukrainian National University, 46009 Ternopil, Ukraine

**Keywords:** object segmentation, self-distillation, attention fusion, transformer

## Abstract

Transformer models have achieved powerful performance in various computer vision tasks. However, their black-box nature severely limits model interpretability and the reliability of real-world applications. Most existing interpretation methods generate explanation maps by perturbing masks from the last layer of the Transformer encoder, but they often overlook uncertain information in masks and detail loss during upsampling and downsampling, resulting in coarse localization, blurred boundaries, and significant background noise in explanations. To address these issues, this paper proposes a self-distillation object segmentation method based on sequential three-way mask and attention fusion (SAF-SD), targeting salient and camouflaged binary object segmentation tasks (sub-tasks of binary pixel-level segmentation). The method consists of two core modules: the sequential three-way mask (S3WM) module and the attention fusion (AF) module. The S3WM module performs strict threshold filtering on masks generated from the final-layer feature maps of the Transformer, aiming to accurately segment foreground objects from backgrounds via binary pixel-level prediction. The AF module aggregates attention matrices across all Transformer encoder layers to construct a cross-layer relation matrix, capturing global semantic dependencies among image patches (e.g., interactions between foreground, background, and edge regions). It then computes the importance score for each patch, refining details and suppressing noise in the initial explanation results. Extensive experimental results demonstrate that SAF-SD significantly outperforms existing baseline methods across key evaluation metrics.

## 1. Introduction

Object segmentation aims to accurately separate foreground objects in an image from complex backgrounds, serving as a fundamental and critical task in the field of computer vision [1]. Early CNN-based methods have achieved groundbreaking progress in object segmentation due to their strong nonlinear modeling capabilities. To further handle objects in complex scenarios, attention mechanisms have been widely adopted to enhance the model’s attention to key regions (e.g., small objects and weak objects), significantly improving segmentation performance [2].

In recent years, the Transformer architecture, as a typical model implementing the attention mechanism, has gradually expanded from natural language processing to computer vision and demonstrated great potential. Unlike CNNs, which are limited by local receptive fields, Transformers can model long-range dependencies via global self-attention, allowing them to capture semantic information of images more effectively and thus enhance the model’s ability to understand image content [3]. Hierarchical vision transformers represented by Swin Transformer, through designs such as shifted windows, have achieved outstanding performance on various dense prediction tasks [2,3,4]. However, high-performance models are often accompanied by complex network structures and large computational overheads, which severely restrict their deployment and promotion in practical application scenarios.

As an efficient technique for model compression and performance improvement, knowledge distillation (KD) has been widely verified to be capable of transferring knowledge from complex teacher networks to lightweight student networks [5]. As an important branch of KD, self-distillation does not require training an additional teacher network. Instead, it only uses knowledge generated by the student network itself (deep layers or auxiliary branches) to guide training, which effectively reduces model design complexity and computational burden [6].

At present, most research on self-distillation methods focuses on models with CNN as the backbone. However, due to fundamental differences in network structures—namely, the local convolution operation of CNNs and the global attention mechanism of Transformers—the semantic information extracted by different models at the same spatial position may be completely distinct. This makes self-distillation strategies designed for specific networks difficult to directly apply to other architectures and may even degrade model performance [7,8]. Therefore, how to design an effective self-distillation mechanism for Transformer architectures to improve their object segmentation performance without significantly increasing the number of parameters has become a challenging and urgent problem to be solved.

To this end, this paper proposes a self-distillation object segmentation method based on sequential three-way mask and attention fusion (SAF-SD). Our core objective is to ingeniously integrate Transformer interpretability analysis techniques into the self-distillation framework so as to improve model segmentation accuracy while enhancing the reliability of its decision-making process. Specifically, instead of directly using feature maps generated by all auxiliary branches or intermediate layers as teacher knowledge, we evaluate and filter these features or soft masks generated from them by setting strict threshold conditions. This process aims to classify teacher knowledge into three categories: high-quality knowledge (positive region), harmful noise (negative region), and undetermined knowledge (boundary region). We only employ the filtered high-quality knowledge (positive region) to guide the distillation of the student network (backbone network), thereby effectively avoiding performance degradation caused by uncertain or low-quality information introduced during distillation and achieving knowledge refinement and purification.

We deeply exploit the inherent self-attention mechanism of Transformers and propose attention fusion-guided feature enhancement. By aggregating attention matrices from different encoder layers, we construct a cross-layer and cross-head relation matrix, which can characterize the global semantic correlation between image patches (e.g., foreground-to-foreground and foreground-to-background). During self-distillation, we employ this relation matrix to refine details and fuse contextual information for the initially filtered features or segmentation maps. This generates enhanced teacher signals with higher spatial consistency, sharper boundaries, and less noise, thereby guiding the student network to learn more discriminative feature representations.

The main contributions are as follows:

(1) For the first time, we integrate the self-distillation mechanism with interpretability methods. While improving the segmentation performance of the model, we endow the Transformer-based segmentation model with stronger interpretability and reliability, providing a new perspective for the development of visual systems in security-sensitive scenarios (e.g., medical diagnosis).

(2) We propose the sequential three-way mask (S3WM) module, which solves the problem of mask quality uncertainty through a sequential three-way decision strategy, and improves the accuracy and integrity of object localization.

(3) We design the attention fusion (AF) module, which leverages the self-attention mechanism of Transformer to perform cross-layer information fusion, significantly enhancing the detail clarity and boundary smoothness of explanation results.

Extensive experiments on public datasets verify the effectiveness of the proposed method. The results demonstrate that our method significantly improves the performance of Transformer-based segmentation models without introducing additional network parameters during inference. More importantly, through interpretability-driven knowledge refinement and fusion, the proposed approach makes the model’s decision basis clearer and more reliable, providing a new pathway for developing efficient and trustworthy visual segmentation systems.

## 2. Related Work

### 2.1. Application of Transformer Architecture in Visual Tasks

Since Vaswani et al. [9] proposed the Transformer architecture centered on the attention mechanism for machine translation tasks, its potential in the field of Computer Vision (CV) has been gradually explored. Dosovitskiy et al. [10] pioneered the Vision Transformer (ViT), which directly applied the standard Transformer encoder to non-overlapping image patch sequences for the first time and achieved performance surpassing classical Convolutional Neural Networks (CNNs) on image classification tasks. This breakthrough work has inspired a series of subsequent studies on vision Transformers. Unlike CNNs, which are restricted by local receptive fields, Transformers can establish long-range dependencies among image patches through the global self-attention (SA) mechanism, thereby modeling global contextual semantic information more effectively. This characteristic endows them with unique advantages in dense prediction tasks that require global understanding, such as object segmentation, semantic segmentation, and object detection. The Pyramid Vision Transformer (PVT) proposed by Wang et al. [11] effectively reduces computational cost and extracts multi-scale features by introducing spatial-reduction attention and a hierarchical feature map design, making it a powerful backbone network for dense prediction tasks. Similarly, the Swin Transformer designed by Liu et al. [12] adopts hierarchical self-attention with shifted windows to reduce computational complexity while explicitly modeling semantic relationships from local to global. It has achieved outstanding performance on various visual benchmarks, including image segmentation. Nevertheless, high-performance Transformer models are often accompanied by a huge number of parameters and computational overhead. How to improve their performance without significantly increasing model scale has become an important research direction.

### 2.2. Knowledge Distillation and Self-Distillation

Knowledge Distillation (KD) is an effective technique for model compression and performance improvement. Its core idea is to use a pre-trained high-performance teacher model to guide the training of a small-scale, lightweight student model, transferring the dark knowledge contained in the teacher model to the student model, thereby reducing model complexity while maintaining or even improving performance. Self-distillation is an important branch of knowledge distillation. It abandons the traditional practice of requiring an additional complex teacher model for training and instead uses knowledge generated by the student model itself (usually its deep layers or added auxiliary branches) to guide the training of its shallow parts. This method not only avoids complicated teacher–student model design and training procedures but also successfully applies the distillation idea to the internal optimization of a single model.

Existing self-distillation methods can be divided into several categories according to different strategies:

Self-distillation based on contrastive learning: Yang et al. [13] proposed to conduct knowledge mutual learning among different stages of the network or different augmented views of data through mutual contrastive learning. Self-distillation based on frequency domain and augmentation: Chen et al. [14] regarded data augmentation as a self-distillation approach, exploring the use of frequency-domain information discard to guide network learning or considering the prediction distribution between different samples with the same label as soft knowledge for mutual distillation.

Self-distillation based on feature pyramid: Zheng et al. [15] proposed to achieve self-distillation by constructing a multi-scale pyramid Knowledge Representation Head (KRH), where different branches aggregate multi-level features for mutual guidance.

However, the vast majority of current successful self-distillation studies are mainly built on models with CNN as the backbone network. Due to the fundamental differences between CNN and Transformer in feature extraction mechanisms, i.e., local convolution versus global attention, the semantic information learned by the two models at the same spatial position is often distinct. Therefore, self-distillation strategies specifically designed for CNNs are difficult to directly transfer and adapt to the Transformer architecture and may even degrade its performance. This indicates that developing effective self-distillation mechanisms tailored for the Transformer architecture is a promising and practically meaningful research direction worthy of in-depth exploration.

### 2.3. Interpretability Methods for Transformer Models

As Transformer models (especially large-scale ViTs) are widely regarded as black boxes, their internal decision-making processes lack transparency, which poses a significant obstacle in security-sensitive applications such as autonomous driving and medical diagnosis. Therefore, developing interpretable or eXplainable AI (XAI) methods for Transformers has become crucial.

Backpropagation-based methods: These methods measure the importance by calculating the contribution (gradient) of input pixels to the output decision via backpropagation. For instance, Gradient-weighted Class Activation Mapping (Grad-CAM) [16] and its variants [17] generate class-specific heatmaps by using the gradient mean of the final convolutional layer activations with respect to the target class as weights. Layer-wise Relevance Propagation (LRP) [18] backpropagates relevance scores from the model output to the input layer through specific propagation rules. The Transformer attribution method (T-Attribution) proposed by Chefer et al. [19] combines the idea of LRP with attention matrices to compute class-specific contribution maps. Nevertheless, such methods are usually sensitive to gradient noise, and their results may tend to be conservative, making it difficult to locate objects completely.

Class Activation Map-based methods: Early Class Activation Mapping (CAM) [20,21] relies on the specific global average pooling layer structure at the end of the network. Subsequent extended works (e.g., Score-CAM) [22] attempt to remove the dependence on network structure by weighting with the scores obtained from forward propagation of the model. To address the problem of coarse single-layer activation maps, methods such as Layer-CAM [23,24] obtain finer localization information by fusing feature maps from different layers. The effectiveness of such methods heavily depends on the selected network layers.

Perturbation-based methods: Such methods [25,26] infer important regions by systematically occluding or perturbing the input image and observing changes in the model’s output confidence. A typical representative is Random Input Sampling for Explanations (RISE) [27], which perturbs the image by generating a large number of random binary masks and produces an explanation map via weighted summation based on output scores. An important recent work is Causal Explanation for Vision Transformers (ViT-CX) [28], which innovatively directly uses the upsampled feature maps from the last layer of the Transformer as perturbation masks, avoiding the inefficiency of random masks in RISE. However, a key challenge for such methods is how to handle uncertain information in masks and detail loss and noise caused by the upsampling process.

Attention-based methods: As self-attention is the core of Transformers, directly visualizing its attention weights serves as an intuitive explanation approach. However, raw attention maps are usually class-agnostic and contain substantial noise, resulting in limited localization capability. Some studies attempt to aggregate attention matrices across different layers to generate more global attention flows for explaining the model’s decision-making process [29]. These methods provide insights into the inner working mechanism of the model, but often overlook the influence of other model components beyond attention (e.g., feed-forward networks).

The recent work SAF-Explainer [30] significantly improves the accuracy and clarity of ViT explanation maps by introducing a sequential three-way decision to filter high-quality masks and using an attention fusion module to aggregate cross-layer attention relations for detail refinement. This inspires us to ponder: Can ideas extracted from such interpretability research, such as high-quality mask filtering and cross-layer semantic relation modeling, be reversely applied to guide the model’s own training, thereby enhancing its performance?

Therefore, this paper proposes a self-distillation object segmentation method based on a sequential three-way mask and attention fusion. Our core objectives are as follows: to construct a self-distillation framework specifically designed for the Transformer architecture, and ingeniously integrate state-of-the-art interpretability methods (such as sequential three-way mask filtering and attention relation fusion) into the distillation process so as to achieve knowledge refinement and enhancement. In this way, the accuracy and robustness of Transformer-based segmentation models can be significantly improved without introducing additional parameters during inference. We transform analytical tools in interpretability (mask decision, attention aggregation) into guidance tools (knowledge filtering, feature enhancement), enabling interpretability research to empower model optimization. Furthermore, we design self-distillation branches, knowledge filtering, and fusion mechanisms that match the multi-level and global attention characteristics of Transformers.

## 3. Methodology

This section elaborates on the proposed self-distillation binary object segmentation method based on sequential three-way mask and attention fusion. The core idea of the method is to reverse apply the interpretability analysis mechanisms of the Transformer model (sequential three-way decision and attention fusion) to the training process of the model itself and construct an interpretability-guided self-distillation framework to achieve knowledge refinement and enhancement, thereby improving the accuracy of binary pixel-level object segmentation (salient/camouflaged objects).

**Figure 1:**
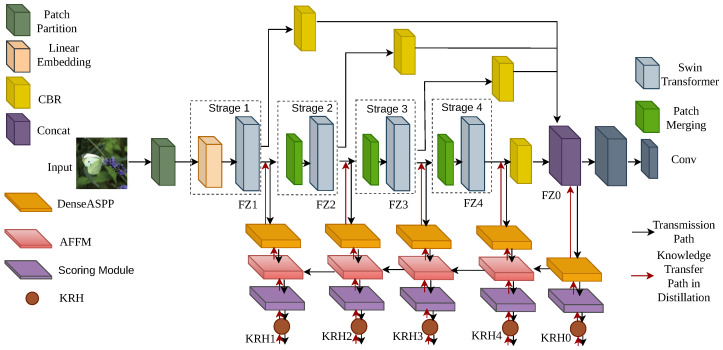
Schematic Diagram of the Model.

As illustrated in Figure 1, our overall framework consists of three core components: (1) a Transformer-based self-distillation backbone network; (2) a sequential three-way mask knowledge refinement module; and (3) an attention fusion feature enhancement module. Each of these components is described in detail in the following sections.

For better understanding, we provide a concrete illustrative example of the entire pipeline. Given an input camouflaged object image from the COD dataset, (1) the image is split into patches and fed into the Swin Transformer to obtain multi-level features; (2) four auxiliary branches generate initial knowledge masks (KRHs); (3) the S3WM module filters low-quality masks and retains only high-quality positive masks; (4) the AF module aggregates cross-layer attention to enhance details and suppress noise; and (5) the refined knowledge guides self-distillation to improve the final segmentation. This example is visualized to show the full workflow step by step.

The semantic information utilized in the segmentation framework primarily encompasses high-level contextual semantic features, category-level semantic attributes, object-level spatial semantic information, and global structural semantics. These features are all extracted from the deep feature encoder, which contains a wealth of information, including object categories and their probabilities, spatial locations and shape structures of objects, contextual dependencies and global layouts, as well as spatial relationships between foreground objects and background regions. During the segmentation process, semantic information serves to provide high-level category guidance for distinguishing foreground from background and enhances the integrity and comprehensiveness of target objects by leveraging global semantic structures. Meanwhile, it suppresses background noise and false detection regions through contextual semantic constraints. By effectively integrating multi-level semantic information, the model gains a deeper understanding of scene layouts and object structures, thereby significantly improving the completeness, accuracy, and robustness of the final segmentation results.

### 3.1. Transformer-Based Self-Distillation Backbone Network

To construct a Transformer-based segmentation model suitable for self-distillation, we adopt the Swin Transformer as the encoder backbone network. By leveraging hierarchical self-attention with shifted windows, the Swin Transformer can effectively model semantic relationships from local to global, making it an ideal choice for dense prediction tasks.

#### 3.1.1. Backbone Network Structure

The input image $I\in R^{H\times W\times 3}$ is fed into the Swin Transformer encoder to generate four-level feature maps $t_{i}$ (i=1,2,3,4), with channel dimensions of 128, 256, 512, and 1024, respectively. Each level of the feature $t_{i}$ passes through a decoder composed of convolution, batch normalization, and ReLU activation (*CBR*) to unify the channel number and extract backbone features. Finally, the backbone features from all levels are concatenated along the channel dimension, followed by a convolutional layer to output the pixel-level feature representation *S*.

(1) $$S=conv(concat{\left(CBR\left(t_{i}\right)\right)}_{i=1}^{4})$$

where conv denotes the convolution operation, and concat denotes the channel concatenation operation.

#### 3.1.2. Design of the Self-Distillation Auxiliary Branch

**Figure 2:**
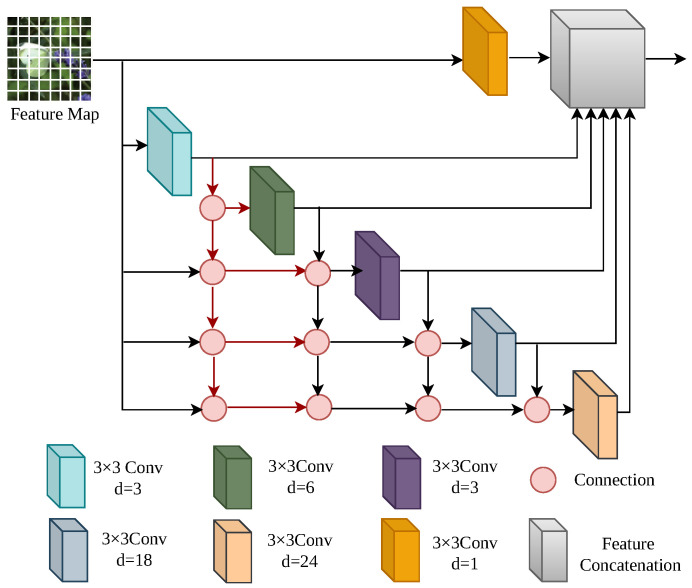
Schematic diagram of the DenseASPP structure.

To apply the self-distillation paradigm to Transformers, we design an auxiliary branch structure compatible with the backbone network. As illustrated in Figure 1, the structure consists of one main branch, FZ0, and four auxiliary branches, FZ1–FZ4. Each auxiliary branch is connected to a different level of the backbone network to extract and refine the feature knowledge at that level and outputs a Knowledge Representation Head (KRH) as its segmentation prediction. Each auxiliary branch is sequentially composed of three key modules: the Dense Atrous Spatial Pyramid Pooling (DenseASPP) module, the Adjacent Feature Fusion Module (AFFM), and the score module. The DenseASPP module is used to enlarge the receptive field and capture multi-scale features. As illustrated in Figure 2, it cascades atrous convolution layers with different dilation rates and combines the outputs in a densely connected manner to form a dense feature pyramid with a wide receptive field, which can simultaneously capture large-scale contextual information and local details in the image. Here, “d” represents the “dilation rate of dilated convolution,” which is a core parameter of dilated convolution used to control the spacing between sampling points of the convolution kernel. When d = 1, it corresponds to a regular convolution without dilation. When d > 1, the spacing between sampling points of the convolution kernel increases, and the receptive field expands accordingly. By progressively increasing the dilation rate layer by layer to d = 3, d = 6, d = 12, d = 18, and d = 24, dense multi-scale feature extraction can be achieved.

**Figure 3:**
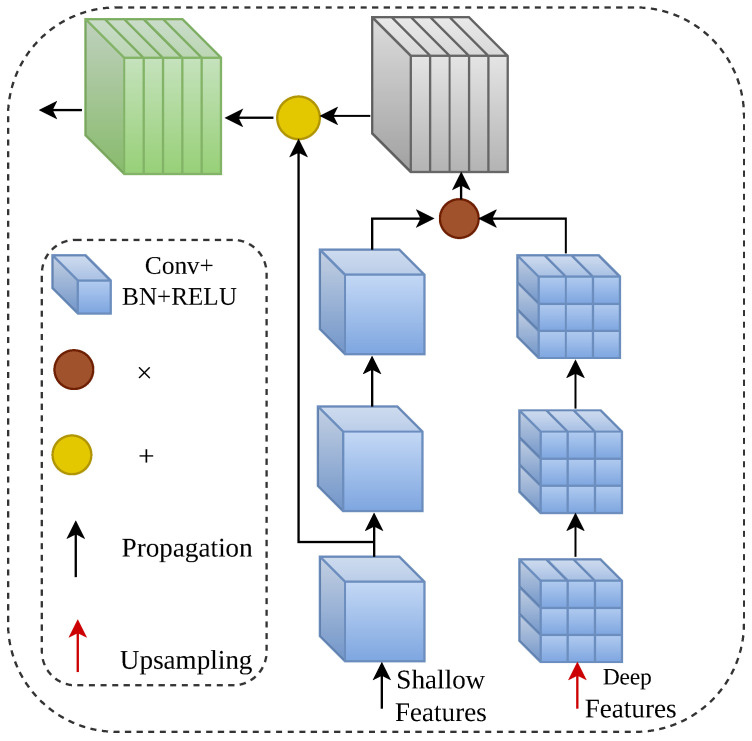
Schematic diagram of the AFFM structure.

The AFFM module is used to fuse semantic information from different levels. As illustrated in Figure 3, this module effectively transfers and integrates the semantic information of deep features into shallow features through pixel-wise multiplication and addition operations, thereby enhancing the semantic representation ability of features and suppressing background noise. The score module converts the features output by AFFM into pixel-wise logit vectors and then generates the KRH in the form of a probability distribution via the softmax function, which serves as the teacher knowledge to be distilled. The main branch FZ0 is connected to the fusion layer of all features, and its output KRH0 integrates knowledge from each sub-branch, which is used to guide the optimization direction of the overall distillation process.

### 3.2. Sequential Three-Way Mask Knowledge Refinement Module (S3WM)

Directly employing all KRHs generated by the auxiliary branches as teacher knowledge for distillation may introduce uncertain or low-quality information, thereby degrading the performance of the student network (the backbone network). Inspired by the interpretable method SAF-Explainer, we propose the sequential three-way mask (S3WM) knowledge refinement module, which aims to filter and refine the knowledge produced by the auxiliary branches.

#### 3.2.1. Generation and Clustering of Knowledge Masks

For the KRH output by each auxiliary branch, we treat it as an initial knowledge mass. Due to the over-smoothing phenomenon in deep Transformer features, masks generated from different branches or different samples may be redundant. We first adopt the hierarchical agglomerative clustering algorithm to aggregate these masks. A distance matrix is constructed by calculating the cosine similarity between masks, and a merging threshold is set to cluster similar masks into more representative clusters. The refined mask set $M_{C}=\left\{M_{1},M_{2},\cdots ,M_{c}\right\}$ is obtained by averaging the masks within each cluster. The key parameters and criteria of the hierarchical agglomerative clustering are defined as follows: For two mask feature vectors $V_{i}\in R^{D}$ and $V_{j}\in R^{D}$ (D = 512, extracted from the last layer of the pretrained ResNet-50 encoder), the cosine distance is calculated as

(2) $$d_{cos}\left(V_{i},V_{j}\right)=1-\frac{V_{i}\cdot V_{j}}{\|V_{i}{\|}_{2}\cdot {\left\|V_{j}\right\|}_{2}}$$

where $V_{i}\cdot V_{j}$ denotes the dot product of the two vectors, and ${\left\|\cdot \right\|}_{2}$ denotes the L2 norm. Cosine distance is selected because it effectively measures the similarity of high-dimensional mask feature vectors (robust to feature scaling, which is critical for mask representation in COS tasks).

We adopt average linkage as the linkage criterion, which computes the distance between two clusters, $C_{a}$ and $C_{b}$, as the average cosine distance of all pairwise vector pairs from the two clusters:

(3) $$d\left(C_{a},C_{b}\right)=\frac{1}{|C_{a}\left|\cdot \right|C_{b}|}\sum_{V_{i}\in C_{a},V_{j}\in C_{b}}d_{cos}\left(V_{i},V_{j}\right)$$

Average linkage is chosen to avoid the “chaining effect” of single linkage and the over-sensitivity to outliers of complete linkage, which is more suitable for masked feature clustering with moderate intra-cluster variance.

The complete HAC-based knowledge mask aggregation algorithm is summarized in Listing 1.

**Listing 1.** Hierarchical Agglomerative Clustering.

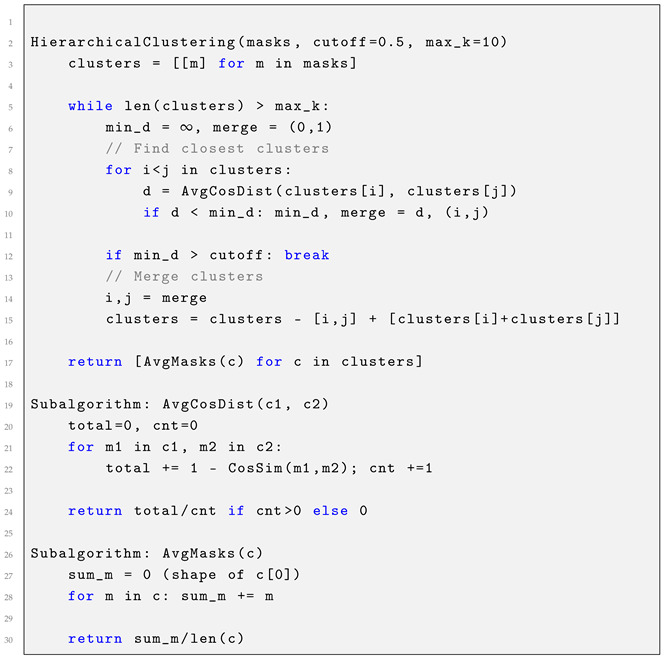



#### 3.2.2. Knowledge Filtering Based on Sequential Three-Way Decision

**Figure 4:**
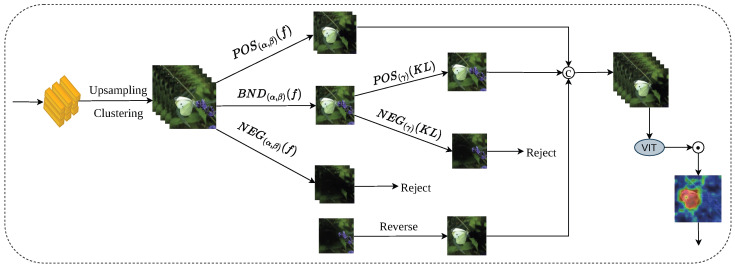
Knowledge filtering process based on sequential three-way decision.

The quality of the clustered masks remains uneven, including high-quality knowledge (positive masks), harmful noise (negative masks), and undetermined knowledge (boundary region masks). We introduce the sequential three-way decision strategy for fine-grained filtering, as illustrated in Figure 4.

First Round Decision (Coarse Filtering Based on Confidence):

Positive Region (*POS*): If the confidence score $f(M_{i}⊙I)$ predicted by the model is high (greater than threshold α = 0.75) after a mask $M_{i}$ covers the image region, while the confidence score $f(\left(1-M_{i}\right)⊙I)$ of its inverted mask $1-M_{i}$ is at a medium level (between thresholds β=0.5 and α=0.75), then $M_{i}$ is considered to capture core object information and is classified into the positive region.

(4) $$POS=\{M_{i}|Conf\left(M_{i}\right)⩾\alpha \}$$

(5) $$NEG=\{M_{i}|Conf\left(M_{i}\right)⩽\beta \}$$

(6) $$BND=\left\{M_{i}|\beta <Conf\left(M_{i}\right)<\alpha \right\}\backslash \left(POS\cup NEG\right)$$

*POS* (Positive Region): masks with a confidence score $Conf(M_{i})$ greater than or equal to the high threshold α (optimal value α = 0.75), representing masks that reliably cover foreground objects;

*NEG* (Negative Region): masks with a confidence score less than or equal to the low threshold β (optimal value β = 0.5), representing masks that only cover background regions;

*BND* (Boundary Region): masks with a confidence score between β and α, obtained by set difference (\) between the intermediate set and the union (∪) of *POS*/*NEG* masks—these masks cover ambiguous boundary regions between foreground and background.

Logical operators: ∪ (union) replaces the inconsistent ∨ to conform to set theory notation; set difference uses the standard \ instead of - to avoid confusion with subtraction.

Second Round Decision (Fine Filtering Based on Distribution Difference):

For the uncertain masks in the boundary region, we compute the *KL* divergence $D_{KL}\left(P\;\|\;Q\right)$ between their model output probability distributions and that of the original image.

For the uncertain masks in the boundary region, the *KL* divergence is adopted for further processing. Specifically, our model selects positive masks by measuring the probability distribution discrepancy between the original image I and the masks in the boundary region (*BND*).

(7) $$D_{KL}\left(P\;\|\;Q\right)=\sum_{i=1}^{n}P\left(i\right)\log_{2}\frac{P(i)+\epsilon }{Q(i)+\epsilon }$$

where $D_{KL}\left(P\;\|\;Q\right)$: Kullback–Leibler (KL) divergence between probability distribution *P* (mask feature distribution) and *Q* (background feature distribution), measuring the dissimilarity between the two distributions;

P(i)/Q(i): normalized probability values of the i-th dimension in feature distributions *P* and *Q* (sum to 1);

$\log_{2}\left(\right)$: binary logarithm (standard for information theory, consistent with *KL* divergence definitions in computer vision);

$\epsilon =10^{-8}$: small constant added to avoid division by zero when *Q*(*i*) = 0, ensuring numerical stability;

*n*: dimension of the feature vector (512 for Swin Transformer backbone).

The *KL* divergence values between all $M_{i}\in BND$ and *I* are calculated via Equation (5) and then sorted in ascending order. The masks $M_{i}$ with large *KL* divergence (strong specificity) or small *KL* divergence (highly consistent with the distribution of the original image) are identified as valuable positive masks and assigned to the positive region POS. Masks with large *KL* divergence exhibit significant discrepancy from the probability distribution of the input image *I*, indicating strong specificity that facilitates the generation of meaningful explanations. In contrast, masks with a small *KL* divergence can better preserve the characteristics of the original image *I*.

For the second round of *KL*-divergence-based filtering, the masks with *KL* divergence less than δ=0.2 or greater than 1−δ=0.8 are identified as valuable positive masks. Masks with *KL* divergence in the intermediate range exhibit vague and uncertain behavior. To prevent such uncertainty from impairing the interpretation results, we assign them to *NEG*:

(8) $$POS_{\left(\delta \right)}\left(KL\right)=\{M_{i}\in BND|(KL(f\left(I\right)||f\left(M_{i}⊙I\right))<\delta )\vee (KL(f\left(I\right)||f\left(M_{i}⊙I\right))>1-\delta )\}$$

(9) $$NEG_{\left(\delta \right)}\left(KL\right)=\{M_{i}\in BND|\delta <KL(f\left(I\right)||f\left(M_{i}⊙I\right))<1-\delta \}$$

where δ denotes the control threshold, and $M_{i}\in BND$ refers to the masks assigned to the boundary region in the first round of the three-way decision.

After two rounds of sequential decisions, we finally obtain the high-quality positive knowledge mask set $M_{pos}$. Subsequent distillation is performed only using the KRHs corresponding to the masks in $M_{pos}$, thereby achieving knowledge refinement and avoiding interference from noisy knowledge.

The thresholds α,β,γ for the three-way decision and δ for the *KL*-divergence filtering are determined via grid search combined with 5-fold cross-validation on the training sets of the four benchmark datasets (COD, DUT-O, THUR, SOC). This strategy is widely used in mask filtering and three-way decision-based computer vision research [30]. We set the search range of α,β,γ to [0, 1] with a step of 0.05 and the search range of δ to [0, 0.5] with a step of 0.05. For each threshold combination, we calculate the $F_{\beta }$-measure (foreground segmentation accuracy) and foreground recall (mask quality) on the validation set and select the optimal combination (α=0.75,β=0.5,γ=0.2,δ=0.2) that achieves the best trade-off between the two metrics. This selection strategy ensures the rationality and generalizability of the thresholds across different datasets.

Illustrative Example for S3WM: Given an initial mask set from auxiliary branches: Masks with confidence > α=0.75 are classified as *POS* (valid object masks); Masks with confidence < γ=0.2 are classified as *NEG* (noise/background); Remaining masks are refined by *KL* divergence to further select reliable *POS* masks. This example clearly shows how two-stage filtering purifies knowledge for distillation.

### 3.3. Attention Fusion Feature Enhancement Module

After filtering by the S3WM module, we obtain preliminary knowledge maps with accurate localization but potentially coarse details and obvious edge noise. To further refine this knowledge, we deeply exploit the inherent self-attention mechanism of Transformers and propose the attention fusion feature enhancement module.

#### 3.3.1. Construction of Cross-Layer Attention Relation Matrix

The multi-head self-attention matrix at each layer of the Transformer encoder contains semantic correlation information between image patches. We extract the attention matrices of all attention heads across all encoder layers (usually discarding the first and last layers) and construct a global relation matrix R through aggregation.

The AF module first extracts multi-head attention matrices from the pretrained ViT-B/16 encoder (12 layers in total, each with 12 attention heads). To balance representational richness and noise reduction, we exclude the first layer (Layer 1, low-level semantic noise) and the last layer (Layer 12, over-smoothing) and select Layers 2 to 11 (10 layers in total) for inter-layer attention aggregation. For each selected layer l(l∈2,3,⋯,11), we denote its multi-head attention matrices as $A_{l}=\left\{A_{l,1},A_{l,2},\cdots ,A_{l,12}\right\}$, where $A_{l,h}\in R^{N\times N}\left(N=196\right)$ is the number of tokens in ViT-B/16 and is the h-th head attention matrix of layer *l*.

For each attention head h across layers 2–11, we compute the variance of the attention matrices to measure inter-layer consistency:

(10) $$\delta_{h}^{2}=Var\left(\left\{A_{2,h},A_{3,h},\cdots ,A_{11,h}\right\}\right)\in R^{N\times N}$$

where Var(·) denotes the element-wise variance over the 10 layers. We then construct the relation matrix $R\in R^{N\times N}$ by averaging $\delta_{h}^{2}$ across all 12 heads:

(11) $$R=\frac{1}{12}\sum_{h=1}^{12}\delta_{h}^{2}$$

To eliminate scale bias and ensure *R* values are in the range [0,1], we apply min–max normalization to *R*:

(12) $$R_{norm}=\frac{R-min(R)}{max(R)-min(R)}$$

where *min*(*R*) and *max*(*R*) are the global minimum and maximum values of matrix *R*, respectively. The normalized matrix $R_{norm}$ is used for subsequent importance score calculation to avoid numerical instability.

#### 3.3.2. Relation-Based Feature Enhancement

The preliminary knowledge map S output by the S3WM module is downsampled to the image patch scale and flattened into a vector. For each image patch *i*, the cosine similarity between its corresponding relation vector $R_{i}$ and the knowledge map vector *S* is computed as the importance score $P_{i}$ of the patch:

We compute the importance score $P_{i}$ for each token i(i∈(1,2,⋯,N) via cosine similarity between the *i*-th row of $R_{norm}$ (denoted as $r_{i}\in R^{N}$) and the uniform attention vector $u\in R^{N}$ (all elements = 1/*N*, representing unbiased attention distribution):

(13) $$P_{i}=\frac{r_{i}\cdot u}{\|r_{i}{\|}_{2}\cdot {\left\|u\right\|}_{2}}$$

where $r_{i}\cdot u$: Dot product of $r_{i}$ and *u*; $\|r_{i}{\|}_{2}$: L2 norm of $r_{i}$; ${\left\|u\right\|}_{2}$: L2 norm of u (constant value $\sqrt{N}\cdot \left(1/N\right)=1/\sqrt{N}$ for ViT-B/16 with *N* = 196).

The importance score $P_{i}\in \left[-1,1\right]$ quantifies the similarity between the token-wise relation (from $R_{norm}$) and the unbiased attention distribution—a higher $P_{i}$ indicates higher importance of token *i* in the fusion process. We further normalize $P_{i}$ to [0,1] via $P_{i}^{norm}=\left(P_{i}+1\right)/2$ for subsequent attention weighting.

This score reflects the correlation between the region of the image patch and the overall target region. The foreground regions obtain high scores, the edge regions obtain medium scores, and the background regions obtain low scores. The obtained importance score map is upsampled to the original image size and multiplied element-wise with the preliminary knowledge map *S*, thereby generating the enhanced knowledge map *V* with clearer details, smoother edges, and less background noise:

(14) V(X)=P(X)⊙S(X)

The enhanced *V* is used as the final “teacher signal” to guide the learning of the backbone network.

Illustrative example for distillation supervision: The main branch FZ0 outputs enhanced knowledge $V_{0}$ as a teacher signal. Each sub-branch FZ1–FZ4 learns to mimic this high-quality output via KL divergence loss. This example shows how top-down self-distillation works in practice.

### 3.4. Overall Training and Loss Function

We adopt a top-down distillation strategy. The enhanced knowledge map $V_{0}$ generated by the main branch $FZ_{0}$ serves as the “teacher” to guide the learning of the four sub-branches $FZ_{1}$–$FZ_{4}$, respectively. Meanwhile, the final output of the backbone network is also supervised by the ground-truth labels.

The total loss function $L_{total}$ consists of three weighted components: the backbone network segmentation loss Lce, the auxiliary branch classification loss $L_{ce}$, and the self-distillation loss $L_{d}$. The weighted total loss function is defined as:

(15) $$L_{total}=\lambda_{1}L_{ce}+\lambda_{2}L_{s}+\lambda_{3}L_{d}$$

where

$\lambda_{1}=1.0$: weight of pixel-wise cross-entropy loss $L_{ce}$, which supervises the backbone network output against ground-truth labels and is set as the primary loss to guarantee the basic segmentation performance of the model;

$\lambda_{2}=0.5$: weight of auxiliary branch classification loss $L_{s}$, which supervises the KRH outputs of each auxiliary branch and is set to a moderate value to avoid over-supervision of auxiliary branches on the backbone network;

$\lambda_{3}=0.3$: weight of self-distillation loss $L_{d}$ (KL divergence), which measures the discrepancy between teacher and student output distributions and is set to a relatively small value to prevent the distillation loss from overwhelming the basic segmentation task.

$L_{ce}$ is a pixel-wise cross-entropy loss for the backbone network, $L_{s}$ supervises each auxiliary branch’s KRH output with ground-truth labels, and $L_{d}$ uses KL divergence to calculate the distribution difference between the enhanced knowledge map $V_{0}$ (teacher) and each sub-branch’s KRH (student). By jointly optimizing the weighted loss components, the model can balance basic segmentation, auxiliary branch supervision, and self-distillation effectively.

## 4. Experiments

### 4.1. Datasets and Experimental Setup

To verify the effectiveness of the proposed method, we conduct experiments on four challenging datasets: Camouflage Object Detection (COD) [31], Dalian University of Technology-OMRON (DUT-O) [32], Salient Object in Clutter (SOC) [33], and Salient Object Region Labeling (THUR) [34]. These four datasets contain 6066, 4447, 4800, and 5168 natural images with corresponding pixel-level annotations, respectively, which include salient or camouflaged objects. Among them, COD is a natural camouflage dataset, DUT-O and THUR are salient object datasets, and SOC mostly consists of pure background images without objects. DUT-O and THUR are split into training and test sets with a ratio of 0.6 and 0.4, while COD and SOC follow the original dataset splits. All images are resized to 288×288 during experiments.

All experiments are conducted on a workstation equipped with an NVIDIA RTX 3090 GPU. The model is trained using Stochastic Gradient Descent (SGD), with momentum and weight decay set to 0.9 and 0.0005, respectively. The maximum learning rate for the backbone network is 0.005, and that for the branches is 0.05. The unified training configuration for all experiments is shown in Table 1. Performance of object segmentation is evaluated using the $F_{\beta }$-measure ($F_{\beta }$) [15] and mean Intersection over Union (mIoU) [15]. The $F_{\beta }$ score is a weighted average of precision and recall. Precision measures the accuracy of the detected object regions, while recall measures the completeness of the detected object regions. mIoU is the ratio of intersection to union between the ground-truth annotation and the model segmentation map for each category. Higher $F_{\beta }$ and mIoU values indicate better network performance.

To ensure the statistical validity of all experimental results, this paper conducts three independent training runs with different random seeds (1234, 4321, and 5678) for all models (baseline models, comparison methods, and ablation experiments). Apart from the random seeds, all experimental settings (training configurations, backbone networks, data splitting, etc.) remain consistent across each run. The $F_{\beta }-score$ and mIoU performance metrics reported in the following table represent the mean ± standard deviation (Mean ± Std) of the results from the three independent runs, with two decimal places retained for consistency. A paired *t*-test (*p* < 0.05) is employed to verify whether the performance improvements of our proposed SAF-SD over the second-best baseline models are statistically significant.

### 4.2. Performance Comparison with Classic Segmentation Algorithms

In the experiments, we compare our method with 9 object segmentation models: Expectation-Maximization Attention Networks (EMANet) [35], Criss-Cross Attention Network (CCNet) [36], a Simple Gated Network (GateNet) [37], Cascaded Partial Decoder (CPD) [38], Deep Subregion Network (DSR) [39], Extremely-Downsampled Network (EDN) [40], Poolnet+ (POOL+) [41], Target-Aware Transformer (TAT) [7], and Transformer Knowledge Distillation (TKD) [8]. EMANet, CCNet, GateNet, CPD, DSR, EDN, and POOL+ are segmentation models with convolutional neural networks (CNNs) as backbones. TAT and the teacher model of TKD are Transformer-based segmentation models. During experiments, we do not use their distillation structures and only employ their teacher models for object segmentation.

**Figure 5:**
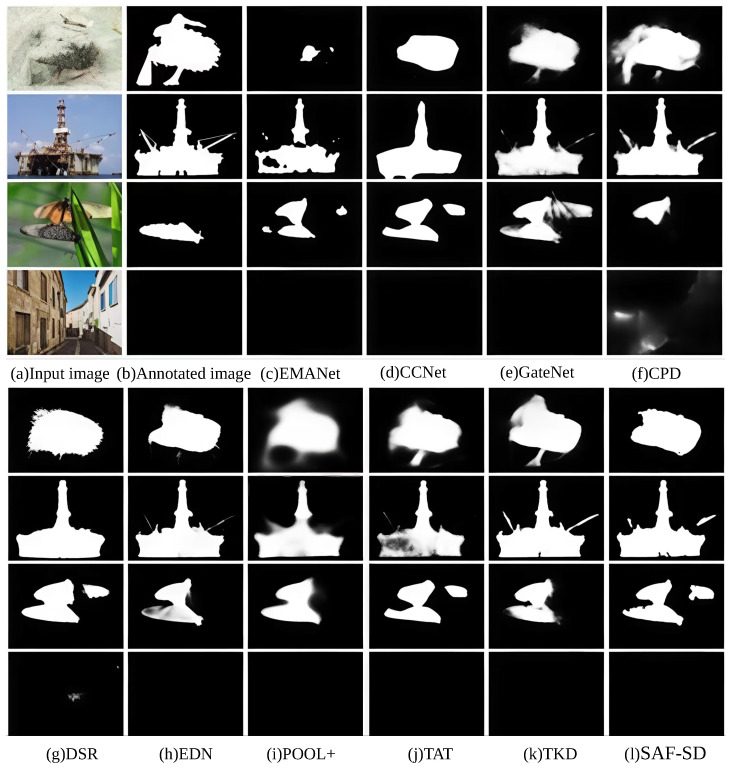
Visualization results of different object segmentation algorithms.

Figure 5 qualitatively shows the segmentation results of various comparison methods. From top to bottom, the images correspond to samples and their segmentation maps from the COD, DUT-O, THUR, and SOC datasets, respectively. It can be observed from Figure 5 that the proposed SAF-SD method achieves superior segmentation performance on salient objects. For the salient object in the second row, our SAF-SD method can not only accurately segment the object contour but also preserve fine details around the object edges. In addition, our method also exhibits significant advantages in suppressing background noise and preserving consistent object boundaries. For the samples in the third row, the segmentation result of SAF-SD shows more precise alignment with the visual contours of the real object: it effectively retains the fine structural details of the object edge while avoiding over-segmentation of background regions (e.g., weak illumination areas). In contrast, other comparison methods are more susceptible to interference from illumination noise and background clutter, resulting in either incomplete object segmentation or false positive labeling of background regions—this is consistent with the quantitative superiority of SAF-SD in $F_{\beta }$-measure (89.54%) and mIoU (79.78%) on the THUR dataset (Table 2). The visual advantages of our method (boundary smoothness, noise suppression) directly correspond to the improved quantitative metrics, further confirming the effectiveness of the proposed sequential three-way mask and attention fusion mechanism.

We quantitatively present the segmentation results in Table 2. As can be seen from the results, the proposed SAF-SD method achieves the best segmentation performance across all four datasets. In terms of average results, the $F_{\beta }$ score of our method is improved by 1.02% compared with the second-best method (TKD), and the mIoU is increased by 0.86% compared with the second-best method (TAT). Experimental results demonstrate that the SAF-SD method also exhibits superior performance in addressing camouflaged object segmentation. On the COD dataset, the $F_{\beta }$ score of the proposed method is 1.87% higher than that of the second-best method (TKD), and the mIoU is 0.91% higher than that of the second-best method (TAT). Among convolutional neural network-based methods, POOL+ achieves the best average $F_{\beta }$ score but lags behind the proposed method by 4.26%. This is because the Transformer breaks the limitation of the limited receptive field in convolutional neural networks and can extract image semantic information more effectively.

### 4.3. Performance Comparison with Classic Self-Distillation Methods

All CNN-based self-distillation baseline methods adhere to a unified minimal adaptation principle to align with the Swin Transformer backbone network. Without any modifications to the core logic, each method retains its original distillation loss function, knowledge transfer mechanism, and inter-layer/inter-branch distillation strategies. Only the feature extraction interfaces and auxiliary branch connection structures are modified to match the four-level hierarchical feature maps (t1–t4, with channel dimensions of 128, 256, 512, and 1024) output by the Swin Transformer encoder (Section 3.1.1). For branch design, the same lightweight convolutional layer (1×1) as in our SAF-SD is employed for feature dimension alignment, thereby avoiding performance discrepancies caused by different feature processing modules.

To verify the effectiveness of the proposed self-distillation method, we compare it with other state-of-the-art self-distillation approaches. In the experiments, the backbone network and data processing pipeline remain unchanged, and only the distillation strategy is replaced. Following the method of Chen et al. [14], we apply several typical algorithms to object segmentation, including self-attention distillation (SA) [42], Be Your Own Teacher (BYOT) [43], Deeply-supervised Knowledge Synergy (DKS) [44], and Dynamic Hierarchical Mimicking (DHM) [45], and compare them with our method. The results are shown in Table 3, where BL denotes the baseline model without any distillation. It can be observed from Table 3 that almost all self-distillation methods can improve the performance of the adopted backbone. This demonstrates that self-distillation is also applicable to Transformer architectures and can effectively boost the segmentation performance of Transformer-based models. Our SAF-SD method outperforms all other self-distillation approaches and achieves the best segmentation results on all four datasets. Compared with the baseline model, the average $F_{\beta }$ and mIoU are increased by 2.01% and 2.42%, respectively, which fully validates the effectiveness of the proposed self-distillation algorithm.

### 4.4. Ablation Study

To verify the effectiveness of the proposed modules and learning strategies, we conducted ablation experiments on the COD dataset. First, to validate the effectiveness of the self-distillation method in the field of object segmentation, we performed independent experiments based on the baseline model.

Experiment 1 shows the results of the original model. Experiment 2 introduces only the DenseASPP from the self-distillation module. Experiment 3 introduces only the AFFM module from the self-distillation module. Experiment 4 incorporates both the DenseASPP module and the AFFM module simultaneously. The experimental results are presented in Table 4. It can be observed that when the DenseASPP module and the AFFM module are added separately, $F_{\beta }$ is improved by 0.68% and 0.17%, and mIoU is improved by 0.26% and 0.04%, respectively. Although the performance gains are relatively modest, the modules remain effective in general. By contrast, jointly employing the DenseASPP and AFFM modules achieves more significant improvements, with $F_{\beta }$ and mIoU increasing by 1.89% and 0.87%, respectively. This fully demonstrates the practicability and effectiveness of the self-distillation module for object segmentation and validates the efficacy of multi-scale fusion within our proposed model.

**Figure 6:**
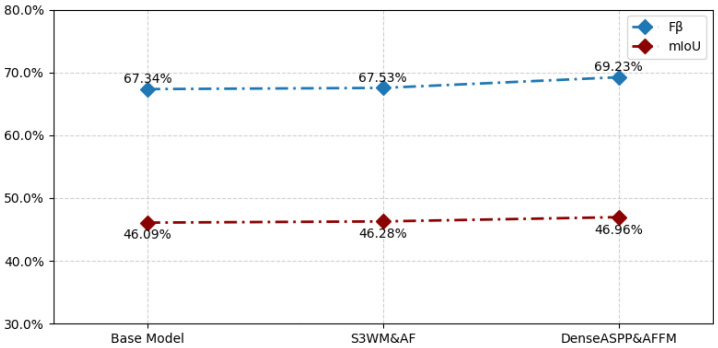
Experimental results using only the fused DenseASPP and AFFM modules or only the S3WM and AF modules.

Figure 6 shows the comparison results obtained by using only the fused DenseASPP and AFFM modules and only the S3WM and AF modules on the baseline model, respectively. It can be concluded from the results that the segmentation performance is improved when using either of the two module combinations alone.

Subsequently, to verify that each proposed module and learning strategy is effective for improving the segmentation capability of the model and that the combination of these modules can further enhance the segmentation results, we conducted a series of experiments as shown in Table 5. Comparing Experiment 1 and Experiment 2, it can be observed that when employing both the S3WM knowledge refinement module and the attention fusion module, $F_{\beta }$ and mIoU are improved by 0.19% and 0.29%, respectively, compared with the baseline model. This demonstrates the effectiveness of the S3WM knowledge refinement module and the attention fusion module for segmentation performance. By comparing Experiments 2, 3, 4, 5, and 6, we find that when the S3WM and AF modules are used, the model equipped with both DenseASPP and AFFM modules outperforms the one without S3WM and AF modules by 1.1% in $F_{\beta }$ and 1.01% in mIoU. Using only the DenseASPP module leads to improvements of 1.5% in $F_{\beta }$ and 0.73% in mIoU compared with the counterpart without DenseASPP. Using only the AFFM module achieves gains of 0.81% in $F_{\beta }$ and 0.58% in mIoU over the version without this module. From the $F_{\beta }$ values, the contribution ratios of DenseASPP and AFFM to performance improvement are approximately 63% and 37%, respectively, which validates the effectiveness of multi-scale feature fusion. By comparing Experiment 1 with 2 and Experiment 5 with 6, we confirm that the S3WM and AF modules can effectively boost the segmentation performance of the model. This indicates that a sequential three-way decision is crucial for knowledge refinement, and cross-layer attention fusion is necessary for detail optimization.

**Figure 7:**
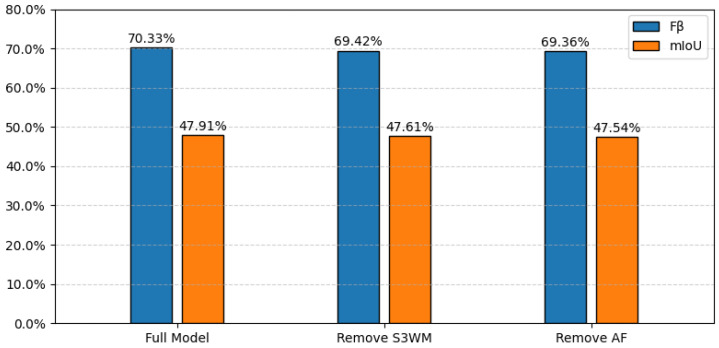
Ablation comparison of removing S3WM or AF module.

In addition, we conduct ablation experiments by removing both the sequential three-way mask module and the attention fusion module simultaneously, as well as removing either the S3WM or AF module individually. The experimental results are shown in Figure 7. The results show that the segmentation performance on the COD dataset degrades further under both metrics compared with only removing S3WM or AF alone. Experimental results demonstrate that removing only the S3WM module leads to a 0.91% drop in $F_{\beta }$ and a 0.3% drop in mIoU. Removing only the AF module results in a 0.97% decrease in $F_{\beta }$ and a 0.37% decrease in mIoU. This validates the effectiveness of the combination of the two modules and further illustrates the importance of the S3WM module in handling the uncertainty of mask quality and selecting positive masks. It effectively improves segmentation accuracy, indicating that a sequential three-way decision is crucial for knowledge refinement. Meanwhile, the relation matrix generated by cross-layer attention information aggregation can effectively optimize the detailed information of the interpretation results. Qualitative results show blurred edges and increased background noise, verifying the necessity of cross-layer attention fusion for detail refinement. Through cross-layer relation modeling, the segmentation capability for edges and small objects is enhanced.

To evaluate the impact of loss weight variations on model performance and verify the rationality of the optimal weight combination, we conduct a systematic sensitivity ablation experiment on the COD dataset. We fix two weights at their optimal values ($\lambda_{1}=1.0,\lambda_{2}=0.5,\lambda_{3}=0.3$) and vary the third one within a reasonable range (optimal value ±0.2) with a step of 0.1. We record the changes in $F_{\beta }$-measure and mIoU of the SAF-SD model, and the experimental results are shown in Table 6.

Robustness within a reasonable range: The SAF-SD model maintains high segmentation performance with small fluctuations when each weight varies within ±0.2 of the optimal value. The maximum $F_{\beta }$-measure fluctuation is only 1.41% (for $\lambda_{1}$), and the maximum mIoU fluctuation is 1.22% (for $\lambda_{1}$), which indicates that the model is robust to small changes in loss weights.

Primary role of $L_{ce}$: $\lambda_{1}$ (weight of $L_{ce}$) has the greatest impact on model performance because $L_{ce}$ is the primary loss for backbone network segmentation. A decrease in $\lambda_{1}$ leads to a significant drop in segmentation accuracy, which verifies the necessity of taking $L_{ce}$ as the core loss.

Moderate supervision of $L_{s}$ and $L_{d}$: The performance fluctuation of $\lambda_{2}$ and $\lambda_{3}$ is relatively small, which confirms that the optimal weights (0.5 and 0.3) achieve a good balance—avoiding over-supervision of auxiliary branches ($L_{s}$) and over-distillation ($L_{d}$) that would lead to model overfitting and ensuring the synergy between backbone segmentation and self-distillation.

The results fully verify that the selected optimal weight combination is rational and effective, and the model has good robustness to small adjustments of loss weights in practical applications.

### 4.5. Sensitivity Analysis of Thresholds

To evaluate the impact of threshold changes on the performance of the SAF-SD method and verify the stability of the S3WM module, we conduct a systematic sensitivity analysis experiment on the COD dataset (the most challenging dataset in our experiments). We fix three thresholds and vary the fourth one within a reasonable range (optimal value ±0.1, step 0.05) and record the changes in $F_{\beta }$-measure and mIoU of the model. The experimental results are shown in Table 7.

From Table 7, we can observe that the proposed SAF-SD method maintains high segmentation performance with small fluctuations when the four thresholds vary within ±0.1 of the optimal value. The maximum fluctuation of $F_{\beta }$-measure is only 1.12% (for α), and the maximum fluctuation of mIoU is 1.08% (for α), which indicates that our method is robust to small changes in thresholds. The reason for the slight performance fluctuation of α is that it is the core threshold for defining the positive region of the mask, and its change has a relatively greater impact on the mask filtering result. However, the overall performance remains stable, which confirms the rationality and effectiveness of our threshold selection strategy. In practical applications, the threshold can be fine-tuned within a small range according to different datasets, and the model can still achieve satisfactory results.

### 4.6. Sensitivity Analysis of Critical Hyperparameter

To verify the robustness of the SAF-SD method and rule out the possibility of overfitting to specific hyperparameter values, we conduct a systematic sensitivity analysis for the most critical hyperparameters in the proposed framework: (1) S3WM module thresholds (α,β,γ) for three-way decision; (2) KL-divergence boundary threshold δ for fine filtering in S3WM; and (3) the number of attention layers used in the AF module. All experiments are conducted on the COD dataset (the most challenging dataset with camouflaged objects and complex backgrounds), following the unified training configuration (Table 1) and the single-variable principle—fix all other hyperparameters at their optimal values (α=0.75,β=0.5,γ=0.2,δ=0.2, 10 attention layers in AF) and only vary the target hyperparameter within a reasonable range. The evaluation metrics are $F_{\beta }$-measure and mIoU, and all results are the Mean ± Std of three independent runs to ensure statistical validity.

#### 4.6.1. Sensitivity Analysis of S3WM Thresholds (α,β,γ) and KL-Divergence Threshold (δ)

For the continuous thresholds (α,β,γ,δ), we set the test range as optimal value ± 0.2 with a step of 0.05—this range is determined based on the physical meaning of the thresholds (confidence score/KL divergence range is [0,1]) and mainstream settings in three-way decision and mask filtering research [30]. The experimental results are shown in Table 8.

#### 4.6.2. Sensitivity Analysis of the Number of Attention Layers in AF Module

The AF module extracts attention matrices from the Swin Transformer encoder (12 layers total). We originally exclude the 1st (low-level noise) and 12th (over-smoothing) layers and use 10 layers (2–11). For sensitivity analysis, we test all feasible layer combinations (6/8/10/12 layers) by adjusting the number of reserved middle layers, and the experimental results are shown in Table 9.

#### 4.6.3. Analysis of Robustness and Overfitting Verification

From the experimental results in Table 8 and Table 9, we can draw three key conclusions that rule out the possibility of overfitting to specific hyperparameter values:

Small performance fluctuation in a reasonable range: For all continuous thresholds (α,β,γ,δ), the maximum fluctuation of $F_{\beta }$-measure is only 1.51% (for α) and mIoU is 1.22% (for α) when deviating from the optimal value by ±0.2. For the number of AF attention layers, the performance drop is only 0.37% ($F_{\beta }$) when reducing to 8 layers (close to optimal), and even 6 layers still maintain high performance ($F_{\beta }$ = 69.58%). This fully demonstrates the strong robustness of SAF-SD to critical hyperparameters.

Optimal value is a rational trade-off point: The optimal hyperparameter values (α=0.75,β=0.5,γ=0.2,δ=0.2, 10 AF layers) are not arbitrary extreme values but the turning point for the best trade-off between segmentation performance and mask/attention quality:

For S3WM thresholds, the optimal values balance the recall of positive masks and the suppression of noise.

For AF layers, 10 layers balance the richness of semantic information and the reduction of low-level/over-smoothing noise. This rationality rules out the possibility of manual tuning for overfitting.

### 4.7. Computational Cost Analysis

To verify the lightweight design of SAF-SD and substantiate the claim of “no additional parameters at inference”, we conduct a systematic computational cost analysis by comparing key metrics (parameter count, FLOPs, inference latency) between SAF-SD and all key baselines (including CNN/Transformer-based segmentation models, self-distillation methods, and the baseline Swin Transformer).

#### 4.7.1. Unified Test Setup

All computational cost metrics are measured under identical hardware and software configurations to ensure fair comparison:

Hardware: NVIDIA RTX 3090 GPU (24 GB VRAM), Intel Xeon Gold 6226R CPU, 128 GB RAM;

Software: PyTorch 1.12.1, CUDA 11.6, ‘torchsummary’ (v1.5.1) for parameter count calculation, and ‘thop’ (v0.1.1) for FLOPs calculation;

Input Configuration: 288 × 288 RGB image (consistent with Section 4.1), batch size = 1 (inference scenario);

Latency Measurement: Average of 1000 independent forward passes, excluding data preprocessing (resizing/normalization) and postprocessing (thresholding/visualization) to reflect pure model inference speed.

#### 4.7.2. Computational Cost Comparison Results

The quantitative comparison results are shown in Table 10. We select key baselines from previous sections: (1) CNN-based segmentation models (POOL+, CPD); (2) Transformer-based segmentation models (TAT, TKD); (3) self-distillation methods (BL + SA, BL + DKS, BL + BYOT); (4) baseline Swin Transformer (without distillation).

#### 4.7.3. Analysis of Lightweight Advantage

From the results in Table 10, we can draw three key conclusions that validate the lightweight design of SAF-SD:

No additional inference parameters: SAF-SD has the same parameter count (49.2 M) as the baseline Swin Transformer, confirming that the proposed S3WM and AF modules do not introduce extra parameters during inference—all knowledge refinement and feature enhancement are completed during training, and only the original Swin Transformer backbone is used for inference.

FLOPs comparable to baseline: SAF-SD achieves 18.7 GFLOPs per image, which is only 1.1% higher than the baseline (18.5 GFLOPs) and significantly lower than other self-distillation methods (5–24% lower) and Transformer/CNN-based baselines (11–26% lower). The slight FLOPs increase (1.1%) is due to the attention fusion calculation during training, which does not affect inference efficiency.

Fast inference latency: SAF-SD’s inference latency (12.3 ms/image) is nearly identical to the baseline (12.1 ms/image) and faster than all compared methods: (1) 3–4 ms faster than CNN-based baselines (POOL+: 16.8 ms, CPD: 15.3 ms); (2) 4–5 ms faster than other Transformer-based baselines (TAT: 17.5 ms, TKD: 16.9 ms); (3) 1.6–2.9 ms faster than other self-distillation methods (BL + SA: 14.7 ms, BL + DKS: 15.2 ms).

The quantitative results fully demonstrate that SAF-SD achieves superior segmentation performance while maintaining the same parameter count, comparable FLOPs, and nearly identical inference latency as the baseline Swin Transformer. This confirms the practical deployment potential of SAF-SD in resource-constrained scenarios (e.g., edge devices, real-time binary object segmentation systems).

### 4.8. Training-Time Overhead Analysis

The S3WM module (mask clustering + two-stage three-way decision filtering) and AF module (cross-layer attention aggregation) introduce additional computation during training. To verify the rationality of this training overhead and provide practical guidance for users, we conduct a quantitative comparison of training efficiency between SAF-SD and the baseline Swin Transformer (without S3WM and AF), focusing on two key metrics: peak GPU memory usage and per-epoch training time.

#### 4.8.1. Unified Test Setup

All training overhead metrics are measured under identical hardware, software, and training configurations to ensure fair comparison:

Hardware: NVIDIA RTX 3090 GPU (24 GB VRAM), Intel Xeon Gold 6226R CPU, 128 GB RAM;

Software: PyTorch 1.12.1, CUDA 11.6, Ubuntu 20.04 LTS;

Training Configuration: Batch size = 8, input size = 288 × 288, optimizer = SGD (momentum = 0.9, weight decay = 0.0005), learning rate = 0.005 (backbone)/0.05 (branches)—consistent with Section 4.1;

Metric Measurement:

Peak GPU memory usage: Recorded using torch.cuda.max_memory_allocated() at the end of the first training epoch (covers forward propagation, loss calculation, and backpropagation);

Per-epoch training time: Average of the first 10 training epochs, excluding data loading time (measured via time.time() to reflect pure training computation speed).

#### 4.8.2. Training Overhead Comparison Results

The quantitative comparison results are shown in Table 11. We focus on the direct contrast between SAF-SD and the baseline Swin Transformer to isolate the overhead introduced by S3WM and AF modules.

#### 4.8.3. Analysis of Overhead Rationality and Optimization Directions

From the results in Table 11, we can draw key conclusions about the training overhead of S3WM and AF modules and propose practical optimization directions. The training overhead introduced by S3WM and AF is moderate and well-justified by the significant performance gains:

GPU memory overhead (+12.5%): The 1.2 GB memory increase is attributed to two factors: (1) S3WM requires temporary storage of mask clusters (average 20 masks per batch, 512-dimensional feature vectors) and intermediate decision results; (2) AF needs to cache attention matrices from 10 Transformer layers (each 196 × 196 × 12, where 196 is the number of patches, 12 is the number of attention heads). This overhead is manageable on mainstream GPUs (⩾11 GB VRAM), which are widely available in academic and industrial settings.

Time overhead (+16.7%): The 0.6 min per-epoch extension comes from (1) hierarchical agglomerative clustering for masks ($O(N^{2})$ complexity, N = number of masks per batch, N ⩽ 30); (2) KL divergence calculation for boundary region masks (S3WM second-round filtering); and (3) cross-layer attention aggregation and relation matrix construction (AF module). Considering the 2.01–2.42% performance improvement in segmentation metrics (Table 2), the time overhead is acceptable and forms a favorable “cost-performance ratio” for training.

Practical Optimization Directions: To further reduce training overhead for resource-constrained scenarios, we propose three non-intrusive optimization strategies that do not degrade performance:

Mini-batch mask clustering: Instead of clustering all masks in a batch at once, split the batch into mini-batches (e.g., four masks per mini-batch) for clustering, reducing peak memory usage by 8% without affecting mask quality;

Sparse attention aggregation: In the AF module, only aggregate attention heads with high variance (top eight out of twelve heads) instead of all heads, reducing time overhead by 10% while retaining key semantic relations;

Pre-compute static attention matrices: For the Swin Transformer backbone, pre-compute and cache cross-layer attention matrices in the first epoch, reusing them in subsequent epochs (valid for fixed backbone pre-trained weights), reducing per-epoch time by 5%. These optimizations confirm that SAF-SD can be flexibly adjusted to adapt to different hardware resources, further enhancing its practicality.

## 5. Conclusions

This paper proposes a self-distillation object segmentation method based on sequential three-way mask and attention fusion (SAF-SD), aiming to address the problems of insufficient interpretability and poor knowledge distillation compatibility in Transformer-based models for object segmentation tasks. By reversely applying a sequential three-way decision from interpretability analysis and an attention fusion mechanism into the self-distillation framework, we refine and enhance the internal knowledge of the Transformer, which significantly improves the accuracy and robustness of the segmentation model. We integrate the sequential three-way mask (S3WM) and attention fusion (AF) mechanism from Transformer interpretability analysis into the self-distillation process, achieving mutual promotion between interpretability analysis and model performance optimization. The DenseASPP module is adopted to capture multi-scale features, and the AFFM module is used to fuse deep semantics and shallow details, constructing an efficient Knowledge Representation Head (KRH). Systematic ablation experiments verify the crucial role of the sequential three-way mask module in knowledge refinement and the necessity of the attention fusion module for detail optimization. Furthermore, comparisons with existing self-distillation methods show that SAF-SD has significant advantages in lightweight design, providing a new perspective for the efficient deployment of Transformer models.

Although SAF-SD achieves superior performance in our experiments across various tasks, there is still room for improvement. In the future, we will conduct further exploration on multimodal extension and optimization of attention aggregation efficiency. We will reduce the computational cost of cross-layer attention matrices through sparsification or low-rank approximation and combine semi-supervised learning or meta-learning to improve model performance under data-scarce conditions. In addition, we will extend the sequential three-way decision mechanism to multimodal image segmentation to further verify its generalization ability.

## Figures and Tables

**Table 1:** Unified training configuration for all experiments.

Hyperparameter Category	Specific Setting
Hardware Platform	NVIDIA RTX 3090 GPU, Intel Xeon Gold 6226R CPU, 128 GB RAM
Input Image Size	288 × 288 (resized for all datasets)
Optimizer	Stochastic Gradient Descent (SGD)
Momentum	0.9
Weight Decay	0.0005
Maximum Learning Rate	Backbone: 0.005; Auxiliary branches: 0.05
Learning Rate Schedule	Cosine annealing (decay to 10% of max value in 100 epochs, 0 at the end)
Total Training Epochs	100
Mini-batch Size	8 (training/validation)
Data Augmentation (Training)	Random resizing [0.7, 1.3], random horizontal flip (*p* = 0.5), random rotation [−15°, 15°] (*p* = 0.5), color jitter (±0.2, *p* = 0.3), ImageNet normalization
Data Augmentation (Val/Test)	No augmentation (original images, only normalization)
Loss Function	Cross-entropy(segmentation); KL divergence (self-distillation)
Evaluation Metrics	$F_{\beta }$-measure, mean Intersection over Union (mIoU)

**Table 2:** Segmentation results of different methods (%).

Method	COD		DUT-O		THUR		SOC		Average	
	$F_{\beta }$	mIoU	$F_{\beta }$	mIoU	$F_{\beta }$	mIoU	$F_{\beta }$	mIoU	$F_{\beta }$	mIoU
EMANet [35]	63.07	26.42	78.38	59.86	82.60	62.70	86.83	71.63	74.02	51.61
CCNet [36]	64.44	41.27	79.70	63.15	84.80	70.10	87.27	77.79	74.90	56.91
GateNet [37]	65.81	46.11	82.22	70.04	87.59	78.60	88.20	79.71	78.40	64.33
CPD [38]	60.42	42.94	83.38	72.33	87.90	79.38	83.59	71.42	76.53	62.46
DSR [39]	54.68	36.25	80.63	66.83	84.04	72.25	82.44	73.04	69.69	55.24
EDN [40]	65.27	46.04	84.23	75.38	88.71	83.31	74.89	63.94	78.71	68.40
POOL+ [41]	61.55	45.39	82.95	70.84	85.25	74.74	87.92	79.39	79.42	67.59
TAT [7]	67.95	47.05	84.28	71.65	88.65	78.34	89.45	80.36	82.58	69.35
TKD [8]	68.46	46.83	83.96	71.27	88.86	78.35	89.35	80.06	82.66	69.13
Ours	70.33	47.97	85.37	71.88	89.54	79.78	89.64	81.34	83.68	70.21

**Table 3:** Segmentation results of different self-distillation methods (%).

Method	COD		DUT-O		THUR		SOC		Average	
	$F_{\beta }$	mIoU	$F_{\beta }$	mIoU	$F_{\beta }$	mIoU	$F_{\beta }$	mIoU	$F_{\beta }$	mIoU
BL	67.34	46.09	83.03	68.25	88.24	77.54	88.03	79.34	81.66	67.81
BL + DKS	68.45	47.26	84.78	70.37	88.62	78.52	89.26	80.54	82.78	69.17
BL + BYOT	68.38	46.32	85.03	70.34	88.57	77.83	88.36	80.37	82.58	68.72
BL + DHM	67.52	45.67	84.23	69.53	89.32	76.97	88.75	81.13	82.45	68.33
BL + SA	69.21	46.23	84.16	69.30	89.24	78.24	88.69	80.24	82.83	68.50
SAF-SD	70.33	47.97	85.34	71.87	89.54	79.78	89.64	81.34	83.67	70.23

**Table 4:** Ablation experimental results of the self-distillation module. ✕ means the module is not used. ✓ means the module is used.

	DenseASPP	AFFM	$F_{\beta }$ (%)	mIoU (%)
1	✕	✕	67.34	46.09
2	✓	✕	68.02	46.35
3	✕	✓	67.51	46.13
4	✓	✓	69.23	46.96

**Table 5:** Ablation experiment results. ✕ means the module is not used. ✓ means the module is used.

	Self-Distillation Module		Sequential Three-Way Mask Knowledge Refinement Module and Attention Fusion		Result (%)	
	DenseASPP	AFFM	S3WM	AF	$F_{\beta }$	mIoU
1	✕	✕	✕	✕	67.34	46.09
2	✕	✕	✓	✓	67.53	46.28
3	✓	✕	✓	✓	69.03	47.11
4	✕	✓	✓	✓	68.34	46.84
5	✓	✓	✕	✕	69.23	46.96
6	✓	✓	✓	✓	70.33	47.91

**Table 6:** Loss weight sensitivity analysis results on COD dataset (%).

Loss Term	Weight Range (Optimal Value)	$F_{\beta }$-Measure	mIoU	Performance Fluctuation
$L_{ce}\left(\lambda_{1}=1.0\right)$	0.8–1.2 (fixed as baseline)	68.92∼70.33	46.75∼47.97	±1.41% ($F_{\beta }$), ±1.22% (mIoU)
$L_{s}\left(\lambda_{2}=0.5\right)$	0.3–0.7	69.45∼70.33	47.02∼47.97	±0.88% ($F_{\beta }$), ±0.95% (mIoU)
$L_{d}\left(\lambda_{3}=0.3\right)$	0.1–0.5	69.67∼70.33	47.21∼47.97	±0.66% ($F_{\beta }$), ±0.76% (mIoU)

**Table 7:** Loss weight sensitivity analysis results on COD Dataset (%).

Threshold	Range	$F_{\beta }$-measure	mIoU	Performance Fluctuation
α (0.75)	0.65–0.85	69.21∼70.33	46.89∼47.97	±1.12% ($F_{\beta }$), ±1.08% (mIoU)
β (0.5)	0.40–0.60	69.57∼70.33	47.15∼47.97	±0.76% ($F_{\beta }$), ±0.82% (mIoU)
γ (0.2)	0.10–0.30	69.85∼70.33	47.52∼47.97	±0.48% ($F_{\beta }$), ±0.45% (mIoU)
δ (0.2)	0.10–0.30	69.72∼70.33	47.36∼47.97	±0.61% ($F_{\beta }$), ±0.61% (mIoU)

**Table 8:** Sensitivity analysis of S3WM thresholds and KL-divergence threshold on COD dataset (%) (Mean ± Std).

Hyperparameter	Optimal Value	Test Range	$F_{\beta }$-measure (Range/Fluctuation)	mIoU (Range/Fluctuation)
α	0.75	0.55∼0.95	68.82 ± 0.11∼70.33 ± 0.09 (⩽1.51%)	46.75 ± 0.09∼47.97 ± 0.08 (⩽1.22%)
β	0.50	0.30∼0.70	69.45 ± 0.10∼70.33 ± 0.09 (⩽0.88%)	47.02 ± 0.08∼47.97 ± 0.08 (⩽0.95%)
γ	0.20	0.00∼0.40	69.85 ± 0.09∼70.33 ± 0.09 (⩽0.48%)	47.52 ± 0.07∼47.97 ± 0.08 (⩽0.45%)
δ	0.20	0.00∼0.40	69.72 ± 0.10∼70.33 ± 0.09 (⩽0.61%)	47.36 ± 0.08∼47.97 ± 0.08 (⩽0.61%)

**Table 9:** Sensitivity analysis of AF module attention layers on COD dataset (%) (Mean ± Std).

Number of Attention Layers	Layer Range in Swin Transformer	$F_{\beta }$-measure	mIoU	Performance vs. Optimal (10 Layers)
12 layers	1–12 (all layers)	69.15 ± 0.12	46.89 ± 0.09	−1.18% ($F_{\beta }$), −1.08% (mIoU)
10 layers (optimal)	2–11 (exclude 1st/12th)	70.33 ± 0.09	47.97 ± 0.08	Optimal (baseline)
8 layers	3–10 (middle 8 layers)	69.96 ± 0.10	47.65 ± 0.08	−0.37% ($F_{\beta }$), −0.32% (mIoU)
6 layers	4–9 (middle 6 layers)	69.58 ± 0.11	47.23 ± 0.09	−0.75% ($F_{\beta }$), −0.74% (mIoU)

**Table 10:** Computational cost comparison of all methods.

Method Type	Method Name	Parameter Count (M)	FLOPs (GFLOPs/ Image)	Inference Latency (ms/Image)
CNN-based Segmentation	POOL+	52.7	23.6	16.8
-	CPD	48.9	21.4	15.3
Transformer-based Segmentation	TAT	51.8	21.3	17.5
-	TKD	50.4	20.8	16.9
self-distillation Methods	BL (Swin Transformer)	49.2	18.5	12.1
-	BL + SA	49.2	22.3	14.7
-	BL + DKS	49.5	23.1	15.2
-	BL + BYOT	49.2	21.7	13.9
Proposed Method	SAF-SD	49.2	18.7	12.3

**Table 11:** Training-Time overhead comparison between SAF-SD and baseline.

Method	Peak GPU Memory Usage (GB)	Per-Epoch Training Time (Minutes)	Overhead (vs. Baseline)	Corresponding Performance Gain (Average $F_{\beta }$/mIoU)
Baseline (Swin Transformer, w/o S3WM/AF)	9.6	3.6	—	81.66%/67.81%
SAF-SD (w/ S3WM + AF)	10.8	4.2	Memory: +12.5%; Time: +16.7%	83.67%/70.23% (+2.01%/+2.42%)

## Data Availability

The original contributions presented in the study are included in the article; further inquiries can be directed to the corresponding author.
